# Bacterial RNA as a signal to eukaryotic cells as part of the infection process

**DOI:** 10.15190/d.2016.17

**Published:** 2016-12-31

**Authors:** Denis Simonov, Simon Swift, Cherie Blenkiron, Anthony R. Phillips

**Affiliations:** Department of Molecular Medicine and Pathology, University of Auckland, Auckland, New Zealand; Department of Surgery, University of Auckland, Auckland, New Zealand; School of Biological Sciences, University of Auckland, Auckland, New Zealand; Maurice Wilkins Centre, University of Auckland, Auckland, New Zealand

**Keywords:** Regulatory RNA, cross-kingdom communication, pathogenicity, innate immunity, RNAi, membrane vesicles

## Abstract

The discovery of regulatory RNA has identified an underappreciated area for microbial subversion of the host. There is increasing evidence that RNA can be delivered from bacteria to host cells associated with membrane vesicles or by direct release from intracellular bacteria. Once inside the host cell, RNA can act by activating sequence-independent receptors of the innate immune system, where recent findings suggest this can be more than simple pathogen detection, and may contribute to the subversion of immune responses. Sequence specific effects are also being proposed, with examples from nematode, plant and human models providing support for the proposition that bacteria-to-human RNA signaling and the subversion of host gene expression may occur.

## SUMMARY

1. *Introduction*
2. ** *Bacterial RNA in the extra- and intra-cellular environment of human cells*
3. ** *Interactions of bacterial RNA with the innate immune system*
4. *Sequence-specific action of bacterial RNA in host cells*
5. *Conclusions*

## 1. Introduction

Cells do not exist in isolation and so cell-to-cell signaling is important in all biological systems; be it a simple community of single-celled organisms or a multicellular organism coordinating information flow between its own cells and those of its microbiota. Through the course of evolution multiple “languages” have been developed for communications between cells that utilize most, if not all, key biomolecules for intracellular and intercellular signaling pathways: proteins, lipids, carbohydrates, and small organic and inorganic molecules. For humans, an important interface is manifested in the cellular and molecular interactions with microorganisms such as both pathogens and symbionts. It is therefore not surprising that these diverse species have evolved mechanisms for interpreting and manipulating the various signaling systems of the other, giving rise to the phenomenon of cross-kingdom communication.

Perhaps our first appreciation of cross-kingdom communication came from the investigation of the molecular mechanisms of bacterial protein virulence factors. The cholera toxin provides a simple example whereby this protein hijacks key intracellular signaling through the second messenger cAMP^1^, leading to a profuse acute diarrhea that is hypothesized to help disseminate the pathogen. A more complex “conversation” has been described between *Salmonella* and its target cells, where the protein effectors delivered by type 3 secretion are primarily responsible for invasion, as well as niche maintenance and dampening of immune responses^2^.

The discovery that bacterial cells communicated to one another via a range of small molecule signals, such as acylated homoserine lactones, in a process termed “quorum sensing” highlighted the possibility that cross-kingdom communication could use a non-protein language^3,4^. confirmed by the finding that the quorum sensing signals used by bacteria to co-ordinate their pathogenic activities could also influence immune responses to the pathogen^5,6^. Moreover, communication is not only from bacterium to target cell, as gene expression is also influenced in bacteria that can intercept intercellular signals deriving from human cells^7^.

Most recently evidence has emerged for a new “language” for communication between host and bacteria that is based on the identification of regulatory RNAs in both prokaryotes and eukaryotes. Our appreciation of the potential roles of RNA is now going beyond the classical designations of messenger, ribosomal and transfer RNAs in the mechanics of translation. Studies have demonstrated that the manipulation of this “riboregulation” is central to the molecular pathogenicity of some viruses^8,9^, and highlighted the hitherto underappreciated role that the subversion of regulatory RNAs could play in progressing infections. The discovery of microRNA (miRNA) signals produced by one cell to influence gene expression in another^10^ has demonstrated RNA as a language of inter-cellular communication identifying them as a potential target for bacterial pathogens.

**Figure 1:**
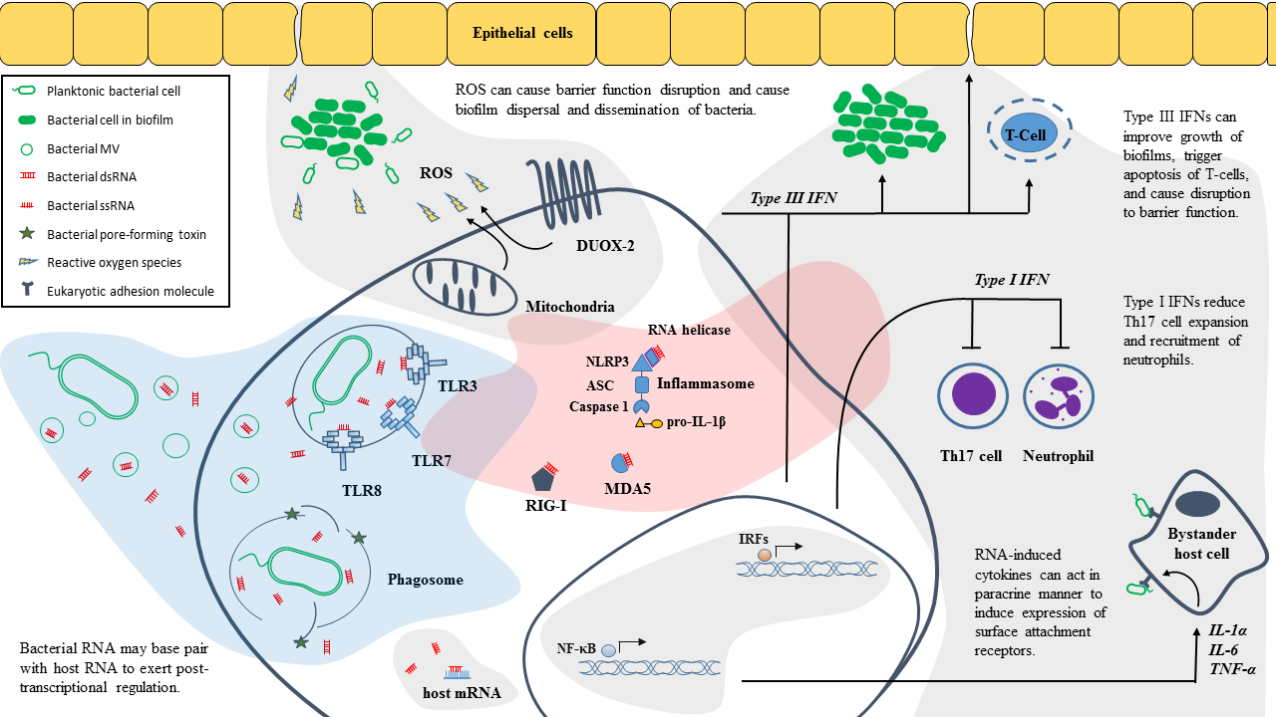
Interactions of bacterial RNA with an eukaryotic host cell a)Entry of bacterial RNA (blue shaded area): Bacterial RNA in double and single stranded forms (red) of extracellular bacteria (green) can enter human cells with MVs, whilst intracellular bacteria can secrete RNA into phagosomes and the cytosol. Bacterial RNA can translocate from phagosomes into the cytosol due to the inherent leakiness of phagosome or when bacterial pore-forming toxins (green stars) disrupt integrity of phagosomes. b) Interactions with the innate immune system (red shaded area): In the endosome, bacterial RNA is sensed by TLR3, TLR7, and TLR8, whilst in the cytosol DExD/H motif helicases such as MDA5 and RIG-I and the NLRP3 inflammasome (via a yet unknown intermediate RNA helicase) can interact with bacterial RNA to trigger downstream signaling cascades. c) Modulation of host cell by bacterial RNA (grey-shaded area): Engagement of innate immune system RNA sensors leads to expression and secretion of type I and type III interferons as well as NF-κB-controlled cytokines which can skew the immune system away from antibacterial response and promote bacterial colonization and dissemination. Activation of the NLRP3 inflammasome leads to Caspase 1 mediated cleavage of pro–IL-1β into active IL-1β. It is postulated that bacterial RNA may also exert post-transcriptional control of human gene expression via sequence-specific interactions with host RNAs. Please refer to the main article for more details and definitions of the abbreviations used.

Today, bacterial RNA is well recognized as an important “pathogen associated molecular pattern” (PAMP) that is involved in human responses to infection. This has been coupled with an appreciation that bacterial RNA is not just a simple uniform trigger of the non-specific immune system, but rather a complex multifaceted signal. For example the differential availability of RNA-recognizing sensors in the host, differences in their subcellular localization and the need to differentiate between ‘self’ and ‘foreign’ RNA define a complex ability of the host to detect intruding RNA and mount a defensive response^11^. In parallel, bacteria can deliver their RNA to the host, for example, using membrane vesicles (MVs) that may protect their cargo while delivering to specific compartments of the host cell^12,13^. In this review, we discuss the current understanding of the role of RNA in human/bacteria interactions (as summarized in**Figure 1**) and provide an outlook for future developments of the field.

## 2. Bacterial RNA in the extra- and intra-cellular environment of human cells

RNA is abundant and a lysed bacterial cell will release about tenfold more RNA than DNA^14,15^. The cells of mucosal barriers and infected tissues will therefore be regularly exposed to RNA from lysed bacteria and other microorganisms. Human tissues and fluids^16-19^ contain high levels of RNases, in the order of several hundred nanograms per milliliter^20,21^, that would be expected to degrade this RNA^22^. However, host miRNA bound to proteins, lipids and lipoproteins is known to be protected from the external RNAses^23^, with the secondary and tertiary structure of some RNA molecules also likely to provide protection from degradation^24^. More recently, advances in small RNA sequencing technology have allowed a detailed analysis of circulating RNAs in mammalian blood, identifying a surprisingly large proportion (ranging from 0.31-11%) as microbial RNA^25-29^. It is therefore probable that some RNA released following bacterial cell death will remain in the immediate environment of the human host.

In addition to the RNA released from lysed bacterial cells, detectable levels of RNA have been reported to be present in supernatants from cultures where most bacteria are viable^30-32^, suggesting that active secretion of RNA may also be at play. Indeed, extracellular RNA from these bacterial populations has been found associated with MVs^12,31,33-35^, as well as in a ‘free’ form^31^. The nano-sized MVs are produced by both gram-positive^36^ and gram-negative bacteria^37^ growing in biofilms, planktonic cultures, inside eukaryotic cells and under a variety of other environmental conditions^38-40^. In many respects, such as size and types of carried molecular cargo, MVs are similar to human exosomes^34^. Human exosomes have a well-established role in RNA communication^41-43^, predominantly through their carriage of regulatory miRNAs, and a similar role for bacterial MVs is beginning to emerge in the literature^12,13^. MVs, like exosomes, protect RNA from degradation as shown by comparisons following RNAse treatment of MVs^12^. The specific protein or other factors that improve the stability of MV-free bacterial RNA are yet to be determined.

A growing body of work is now available to support various mechanisms of outer membrane vesicle production by Gram-negative bacteria that involve budding from the outer membrane, induction by stress responses and some selectivity in the composition of the MV cargo^44-46^. An alternative mechanism for the formation of MVs via explosive cell lysis has recently been identified for *Pseudomonas aeruginosa*^35^. Strains that encode a prophage endolysin (A0629) can undergo an explosive cell lysis event in response to exogenous stress and produce MVs through vesicularization of membrane fragments. This process allows capture of cellular components released into the extracellular space, including the incorporation of RNA into the MVs. It is yet to be determined if explosive cell lysis under non-stress conditions is a programmed cell death pathway induced by “altruistic suicide”^47^ to release key nutrients to other bacteria as a “colony public good”, or if it is the result of stochastic expression of the A0629 endolysin or if it could be part of a regulated virulence program. It will be of interest to determine if this process occurs in other species of bacteria, as genes with high similarity to A0629 can be found in the genomes of several other bacterial genera^35^.

Free RNA added to the medium of cultured cells can induce responses that favour bacterial colonization and immune evasion^32^. However, the relevance of this role in an infective setting is unclear as RNA released from bacteria into the extracellular environment probably has a long way to travel before it can influence the activity of host cells. Mechanisms of bacterial MV entry into host cells have been widely studied^48-56^, although it has not yet been shown which mechanism(s) are involved in the uptake of RNA-carrying MVs. Bacterial MVs are a heterogeneous population and we speculate that RNA-carrying MVs may represent only a fraction of the total MV population. At the same time, the mechanism of host entry by a bacterial MV, and consequently the targeting and fate of its cargo, is likely to be different for various MV subpopulations and influenced by MV size, surface molecules etc. In cases where the intracellular delivery of bacterial RNA has been studied, the evidence obtained currently suggests it is necessary for the induction of responses associated with exogenous RNA sensing^57,58^ (discussed in the section “Interactions of bacterial RNA with the immune system” below).

An alternative route for bacterial RNA to enter human cells is via release from intracellular bacteria. *Listeria monocytogenes* is a model organism for the study of intracellular bacteria/host interactions^59^. When the RNA of live *L. monocytogenes *was labelled with a modified nucleotide, 5-ethynyluridine (5EU), and these bacteria were allowed to infect the human monocytic cell line THP-1 the visualization of bacterial RNA by chemically attaching a fluorophore to the 5EU-RNA revealed it in an extra-bacterial localization in the host cell cytosol^60^. Further, no evidence of cytoplasmic, extra-bacterial RNA was found for THP-1 cells infected with 5EU-labelled *L. monocytogenes *lacking the SecA2 auxiliary protein secretion system^53^, suggesting that the bacterial RNA is actively secreted, possibly in association with a protein chaperone, rather than being a by-product of bacterial lysis.

In a related report, RNA released from bacteria that themselves remain trapped in the phagosome can also exert an effect. In the case of *Borrelia burgdorferi, *this occurs via RNA interaction with the endosomal TLR8 to induce transcription of IFN-β^61^. In other instances, RNA from the phagocytosed bacteria can translocate into the cytosol from phagosomes that are intrinsically leaky^62^, or made leaky by the actions of bacterial pore-forming toxins^63^.

Overall, the reported findings from the last several years demonstrate that bacterial RNA is a common component in the environment of many human cells. Additionally, current evidence suggests that release of RNA by bacteria is not a simple by-product of bacterial cell death, but an active, and possibly, selective process. The variety of mechanisms involved in the secretion of RNA from bacterial cells, its stability in host extracellular fluids, its association with transport systems for intra- and inter-species signaling such as MVs, and its secretion into host intracellular compartments by live intracellular bacteria suggest that RNA may be used by bacteria as a currently underappreciated virulence factor. Which pathways are engaged by bacterial RNA will likely depend at least in part, on where inside a human cell bacterial RNA is delivered. Evidence to date suggests that bacterial RNA can be delivered into human cytosol^30,59,64,65^, endosomal and phagosomal compartments^66,67^ with one study reporting delivery of bacterial MV RNA into human cell nuclei^13^. Thus, current research has focused on the interactions of bacterial RNA with cytosolic and endosomal receptors of the innate immune system and on the investigation of possibilities for bacterial RNA to affect human gene expression via post-transcriptional mechanisms in a sequence-dependent manner. Key recent discoveries from each of these fields are discussed in the following sections of this review.

## 3. Interactions of bacterial RNA with the innate immune system

Entry of bacterial RNA into a host eukaryotic cells is sensed as a danger signal by receptors of the innate immune system. In the endosome, RNA can be recognized by TLR3, TLR7, and TLR8. TLR3 senses long (>39bp) double stranded RNA (dsRNA)^68^, while TLR7 and TLR8 sense degradation products of single stranded RNA (ssRNA). All three TLRs recognize RNA in a sequence-independent manner, although there is some evidence for preferential recognition of RNA rich in some nucleotides or modified nucleotides^69,70^. It has been proposed this relates to the observation that some pathogens display a greater proportion of certain types of nucleotides in their RNA sequences^71^. Furthermore, for at least TLR8, the key descriptors for RNA recognition are not yet certain, as THP-1 cells (a cultured monocyte line) challenged with enterococcal RNA only responded (measured as IL-12 production) when 23S and 16S rRNA, but not when mRNA, were applied^72^.

In the cytosol, some of the key sensors of bacterial RNA are two helicases of the DExD/H motif family, namely RIG-I and MDA5, which detect 5’ triphosphorylated short dsRNA and long dsRNA, respectively^73^. RIG-I binds blunt ends of dsRNA displaying a 5’ triphosphate moiety whilst MDA5 molecules bind RNA independently of its terminal structures. Additionally, the NLRP3 (NLR family, pyrin domain-containing) inflammasome has recently emerged as an important cytosolic sensor of bacterial RNA^57,74,75^. It is a signaling complex that consists of the sensor molecule NLRP3, the adaptor protein ASC and caspase 1, which, in addition to bacterial RNA, senses a variety of endogenous and pathogen-associated molecules (pore-forming cytotoxins, ATP, uric acid^76^).

Induction of the innate immune sensors for bacterial RNA can lead to secretion of type I and type III interferons, and pro-inflammatory cytokines such as TNF-α, IL-1α, IL-1β, IL-6, IL-8, IL-10, and IL-12^14,15,32,58,59,61,72,77-82^. However, which RNA sensors are engaged and what host effector molecules are secreted is cell type specific. For example, IFN-α secretion is induced in murine plasmacytoid dendritic cells upon DOTAP-mediated transfection of *Escherichia coli* RNA but not in conventional dendritic cells (cDCs)^80^. IFN-β is secreted by murine cDCs in response to transfection with RNA from Group B^15^ and Group A streptococci^78^. However, in bone marrow-derived macrophages IFN-ß only responded to transfection of DNA, and not RNA, of Group A streptococci. These differences in responses to bacterial RNA reflect differential availability and use of molecular RNA sensors in different cell types. Differences in host cell responses to the type of RNA (tRNA, rRNA, mRNA etc) can also reflect variations in post-transcriptional modifications of a given prokaryotic RNA molecule across various species^80,83-85^. For example, when polyadenylated, prokaryotic mRNAs have a shorter poly-A tail than their eukaryotic host mRNAs, and as such can be readily detected by TLR7^69^. Additionally, the 2’O-methyl guanosine modification status of tRNA at the conserved G18 residue can determine TLR7 activation. For some pathogenic bacteria, where G18 is unmodified, the induction of type I interferon and pro-inflammatory cytokines is seen in mouse dendritic and human peripheral blood mononuclear cells, whilst modified tRNA from non-pathogenic *E. coli* Nissle 1917 and *Thermus thermophilus* does not^86^. Finally, the cellular localization of the pathogen (cytosolic or phagolysosomal for instance) determines which sensors of bacterial RNA are engaged^11,22^.

Studies of innate immune responses to bacterial RNA have mostly focused on the identification of host cell receptors for bacterial RNA and downstream signaling cascades. Minimal research has been done to investigate how bacteria could use their RNA to manipulate innate immunity to their advantage. Notably, many of the effector molecules secreted by host cells in response to detection of bacterial RNA are also secreted in response to viral RNA. In fact, the RNA sensors discussed above were originally discovered as sensors of viral infections. In the last decade they have also been validated as sensors of bacterial RNA^74,80,87^. Studies of secondary bacterial infections that develop as a complication of viral infections, could therefore provide some insights into the relevance of RNA-induced cytokines on the progression of bacterial infections. Interestingly, it has been observed that, although common host mediators are induced, antiviral and antibacterial responses can frequently be at odds with one another^88^. This provides an opportunity to speculate that bacteria might use interactions of its secreted RNA with host innate immune sensors to its advantage by skewing the immune system towards antiviral responses. In evolutionary terms, it is possible to imagine how such a mechanism for manipulating the human host could have evolved in bacteria. The human microbiome includes not only bacteria but multiple other types of organisms including viruses, fungi and protozoa^89^. Bacteria/human cross-kingdom interactions are, therefore, a product of dynamic coevolved relationship between the various organism of the microbiome and the immune system. It is therefore possible that bacteria might have evolved to utilize elements of human/virus interaction to their advantage even in the absence of viral co-infection. In this case, we hypothesize that the secretion of bacterial RNA and its detection by human cells could create a beneficial environment for bacteria, similar to that of viral-bacterial co-infection.

The strongest evidence for the beneficial effects of RNA-induced cytokines on a bacterial infection process comes from studies of type I interferons. Whilst induction of type I interferons can lead to host protection and elimination of the pathogen during infections with *Chlamydia trachomatis*^90^, *Salmonella enterica*^91^, *Cryptococcus neoformans*^92^, Group B streptococci, *Streptococcus pneumoniae* and *E. coli*^93^, there is evidence that indicates that type I interferons can also inhibit host defense against other bacteria^94,95^. For example, pathogen-induced production of type I interferons has been suggested to contribute to the pathogenesis of *Mycobacterium tuberculosis* infections^96^, and the dissemination of *B. burgdorferi* during the early stages of infection^97^ where TLR7-dependent recognition of *B. burgdorferi* RNA is necessary for interferon-α production^77^.

Evidence that bacteria could benefit from interferon-induced skewing of the innate immunity towards antiviral responses also comes from investigations of the activation of T helper 17 (Th17) cells. The Th17 pathway has a critical role during infection with extracellular bacteria^98^. IL-17 and IL-22 are hallmark cytokines of Th17 cells, and have been shown to promote clearance of bacteria through the recruitment of phagocytes and the induction of antimicrobial peptides (AMPs)^99^. Induction of type I interferon production by epithelial cells in response to viral infections skews the immune status towards an antiviral phenotype and attenuates type 17 immunity against such bacterial pathogens as *E. coli* *and P. aeruginosa*^100^, *S. aureus*^101^ and *S. pneumoniae*^102^. The identification of bacterial communications that direct immune responses away from antibacterial activity have also been a reported feature of the response to some bacterial quorum sensing signals^5^.

Interactions of bacterial RNA with innate immune sensors could also serve a beneficial function to bacteria via mechanisms not involving interferon signaling. Transfection of bacterial RNA has been shown to induce reactive oxygen species (ROS) production via mechanisms involving NADPH oxidase and the mitochondrial transport chain^58^. ROS, in turn, can disrupt epithelial barrier function. Specifically, it has been reported that, in polarized airway epithelial cells, poly I:C (a synthetic mimic of dsRNA) signals through a recently discovered cytosolic dsRNA receptor Nod-like receptor X-1 (NLRX-1) and stimulate NADPH oxidase 1 (NOX-1) and mitochondrial ROS production to cause reactive oxygen species (ROS) dependent epithelial barrier function disruption^103^. A poly I:C challenge also disrupted endothelial barrier function by causing downregulation of the mRNA for claudin-5, a key endothelial tight junction protein. The exact mechanism is unknown but appears to involve a TLR3-TRIF-NF-kB signaling pathway^104^.

Bacterial RNA engagement with host cell RNA sensors to induce reactive oxygen production can lead to dispersion and dissemination of bacterial biofilm cells. For example, bronchial epithelial cells have been shown to express dual oxidase 2 (Duox2) in response to poly I:C and IFN-γ treatment^105^. Duox2 is located in the plasma membrane and can secrete H_2_O_2_ directly into the extracellular milieu^106^. Biofilm bacteria, when exposed to oxidative stress, can initiate a dispersal response^107^ to release free-swimming planktonic bacteria. Whilst induction of H_2_O_2_ production as an immune response can be viewed as a negative event for bacteria, both biofilm-associated and planktonic bacteria express antioxidant enzymes to resist H_2_O_2_ killing^108^. Additionally, planktonic bacteria dispersed from biofilms are often as resistant to killing by antimicrobials as their biofilm counterparts^109^. An example of how this process can lead to worsening of clinical outcomes can be found in cystic fibrosis patients with chronic *Pseudomonas aeruginosa* infections. It has been reported in this setting that a viral infection causing mild oxidative stress via activation of Duox 2 leads to dispersal of planktonic bacteria from established lung biofilms, increased transmigration of planktonic bacteria from the apical to basolateral surface of mucociliary-differentiated airway epithelial cells, increased planktonic bacterial and therefore the acute symptom burden^110^.

In an infection, the RNA of pathogenic bacteria may subtly manipulate the host innate immune response to activate inappropriate defense responses that can ultimately favour bacterial survival. In other instances, bacterial mechanisms for disguising their RNA using post-transcriptional modifications can become of importance^79,85,86^. Bacteria can also use non-RNA virulence factors to interfere with signaling cascades downstream of RNA-sensors. An example of this was recently reported that showed production of IFN-β induced by TLR8-mediated sensing of *S. aureus* ssRNA, is antagonized by TLR2 signaling activated by *S. aureus* lipoproteins^111^. Future detailed investigations of the molecular interactions between bacteria and innate immunity that involve bacterial RNA may eventually provide a better understanding of what stimulates a productive immune response and identify opportunities for developing novel therapeutic strategies. Overall, these findings confirm that detection of bacterial RNA by the innate immune system is not always as simple as an immune surveillance ‘hit’ leading to responses that eliminate bacterial pathogens.

## 4. Sequence-specific action of bacterial RNA in host cells

Bacterial RNA is seemingly not just a ligand for sequence-independent RNA receptors, but is starting to be appreciated for its potential to act in a sequence-specific manner to regulate gene expression at the post-transcriptional level. It is estimated that ∼60% of the human protein coding genes could be subject to regulation by their regulatory miRNAs^112^ offering a large target for bacteria to manipulate host cells to their advantage using the same endogenous RNA-inhibition (RNAi) machinery. The use of RNA as an effector molecule by directly targeting host RNA may offer the advantages of suppressing the expression of mediators of immunity before they can exert any antibacterial effects i.e. prior to the production of the protein effector itself.

It is interesting to speculate on how a bacterial pathogen’s RNA might regulate the host in a sequence-specific manner. The first question to ask is whether a bacterial RNA could bind to a host mRNA? There are differences in the specific mechanisms of action of known non-coding RNAs between human and bacterial cells, and RNA regulation in the eukaryotic host is substantially more complex^113^, but there are several commonalities. Specifically, regulation through direct hybridization between the regulator and the target RNA, mediated by complementary base-pairing, is employed by both kingdoms. For example, both bacterial regulatory RNAs and human miRNA are double-stranded which helps to stabilize the RNA molecule and allows correct orientation of the regulator in relation to the potential target mRNA and accessory proteins^114^, such as AGO2 in humans and Hfq in bacteria. Both human and bacterial regulatory RNAs tend to have a single stranded ‘seed’ region that is devoid of secondary structure^115^ to allow perfect antisense binding to mRNA targets. Additionally, both miRNAs and bacterial small RNAs (sRNAs) can have varied levels of complementarity with their target RNAs, which in combination with the short seed sequence allows the binding of a single miRNA/sRNA with multiple target mRNAs^116^.

The second question to ask is whether naked bacterial RNA could hijack the hosts own protein machinery to allow it to work in a regulatory manner? Interestingly the eukaryotic RNAi machinery appears to have been put together from various prokaryotic sources (the helicase domain of Dicer and AGO from archaea, the RNase domain of Dicer from bacteria, and RNA-dependent RNA polymerase from bacteriophages)^117^. Furthermore, bacterial sRNA-binding Hfq is an ortholog of eukaryotic Lsm proteins^118^ which also act as RNA chaperones to aid in splicing and degradation. Preliminary studies also support that exogenous RNA, from viruses^118^ and bacteria^25,119^ can bind to host AGO proteins, key proteins in the RNAi machinery.

With laboratory studies being technically challenging and to date limited, the prediction of what targets a bacterial RNA may have relied on computational modelling. These models require many assumptions to be made and as such later wet-lab validation is vital. Most such studies begin by identifying RNA molecules in the non-human organism that could function as a miRNA mimic when transferred into human cells. Such studies have had success in models for cross-kingdom RNA signaling where both organisms have endogenous miRNAs such as the plant *Arabidopsis thaliana *and humans, and the nematode *Heligmosomoides polygyrus *and mice^120,121^. Bacteria, however, do not have eukaryotic-like miRNA and their known non-coding RNAs range from 40 to 500 nucleotides in length^122^, which is in contrast to the average length of a human miRNA of about 22 nucleotides^123^. It is possible that long bacteria RNAs are processed into functional fragments. Shmaryahu et al.^124^ developed a high throughput bioinformatics pipeline, using the assumption of RNA fragmentation, to analyze all genes in a bacterial genome for their potential to produce RNA transcripts with secondary structures containing double-stranded regions that, through processing, could give rise to miRNA-like fragments. An analysis was made of 448 bacterial genomes, identifying on average 15 putative miRNA-like sequences per organism that could bind human mRNA. The authors validated the *in silico* analyses by synthesizing mimics of three predicted bacterial RNA-derived ‘miRNAs’ and transfected them into the human HEK293 cell line. The mimics represented a sequence derived from *Arcobacter butzleri* strain RM4018, a close taxonomic relative of *Campylobacter jejuni* and *Helicobacter pylori*^125^, with complementarity for the human DEK oncogene mRNA, a sequence from *Burkholderia vietnamiensis G4* with complementarity for the transcript variant 2 mRNA of the human tumour suppressor PTPRJ (protein tyrosine phosphatase receptor type J) gene and a putative sequence from *Burkholderia mallei* with complementarity for the human NFKBIL1 (nuclear factor nuclear factor kappa-light- chain-enhancer of activated B cells) mRNA. Following transfection, mRNA levels of the predicted target genes were determined by RT-qPCR as reduced in expression. This study provides support for the hypothesis that bacterial RNA sequences could potentially target human mRNA, however, the study did not investigate if these putative sequences exist in nature or if they could be transferred from bacteria into human cells to function in the predicted manner.

Koeppen et al.^12^ came closer to describing a bacteria-to-human RNA communication system by demonstrating natural transfer of endogenous bacterial short RNA species from* Pseudomonas aeruginosa* into human host cells via MVs, whilst also demonstrating a reduction in protein levels of several kinases whose mRNAs were predicted to be targeted by one of the bacterial MV sRNAs. Specifically, Koeppen and co-authors identified that sRNA52320, a 24-nucleotide tRNA fragment, is transferred into human cells upon exposure to MVs and that it decreases translation of MAP3K7 and MAP2K4 (kinases in the LPS-simulated MAPK signaling pathway) with subsequent reduction in MV-induced host cell secretion of IL-8.

Overall, these studies have provided early indications that bacterial RNA could act in human cells in a sequence-specific manner to exert post-transcriptional effects on gene expression. Studies of two model systems *Caenorhabditis elegans* and *Arabidopsis thaliana* have begun to generate interesting hypotheses and scientific debate based on the biology of RNA in the interactions of *C. elegans* with dietary *E. coli* and *A. thaliana* with the mould *Botrytis cinerea*. However, some caution in the interpretation is required here as these two hosts have systems to allow signal amplification of exogenous RNA via RNA-dependent polymerases^126,127^. This mechanism, overcomes a potentially contentious issue in the bacteria-to-human signaling field, concerning whether**the amount of bacterial RNA transferred is enough to have a function in the host cells.

Alterations in physiological functions were observed in *C. elegans *fed with *E. coli *overexpressing the non-coding RNAs OxyS (an oxidative stress response regulator) and DsrA (an acid stress response regulator)^128^. In bacteria over-expressing OxyS, a negative foraging effect was observed in the nematodes, which preferred to feed on *E. coli *not over-expressing OxyS when given a choice. The nematodes did not exhibit the repulsion effect when only fed with the OxyS over-expressing strain. Computational analysis of the *C. elegans* genome identified the *che-2* chemosensory gene as a possible target for a sequence-specific interaction with OxyS. Down regulation of CHE-2-GFP fusion expression was visibly seen in *C. elegans* fed on the OxyS over-expressing strain when compared to wild type K12 *E. coli *controls.

Interpretation of these results above led the authors to a hypothesis of environmental RNAi, with OxyS from the diet bacteria suppressing the ability of *C. elegans* to find them. It was shown that the transferred OxyS required the host RNAi pathway, specifically proteins ALG-1 and RDE-4, to function to repress the target CHE-2 protein^128^. The fact that other RNAi genes were described as dispensable^128^ raised questions regarding the exact biological mechanism eliciting the gene expression changes and behavioural responses observed^129^ and this second, independent study, was unable to support the findings of the original study. By using small RNA-sequencing they were unable to find evidence of OxyS RNAs in fed *C. elegans* that could capably bind to the 17nt interaction site on the *che-2* target mRNA and were unable to validate regulation of the target mRNA itself^129^. Differences between these two study’s findings^128,129^ could in part be explained by the use of different strains of dietary bacteria, and differences in feeding experiment protocols, such that the role of OxyS RNA might only manifest when the nematode has to make a choice about its food. Further investigations are needed to explain the action of OxyS RNA in *C. elegans*, and the story to date highlights the power we now have with RNA sequencing to test complex sequence-specific cross-kingdom communication hypotheses.

RNA sequencing was also a key technique used to determine the potential role of fungal small RNAs in the infective processes of *B. cinerea *using the model plants *A. thaliana* and *Solanum lycopersicum*^130^. *B. cinerea *sRNAs involved in pathogenicity were identified in infected plants, and a simple bioinformatic approach identified sRNAs with miRNA-like structures that were predicted to suppress 4 genes, by perfect antisense binding, all with roles in plant immunity. Ectopic expression of three of these sRNAs in *A. thaliana* left the plant with an increased susceptibility to infection. Importantly, and unlike the situation with *C. elegans*^129^, the microbial sRNAs were shown to bind to host AGO1 within the RNAi machinery, and *A. thaliana *strains in which AGO1 is mutated exhibit reduced susceptibility to *Botrytis *infection. Finally, they demonstrated that *B. cinerea *Dicer mutants lacking the ability to process sRNAs from longer transposon RNAs were less infective. The challenge now is to identify and explain sequence-specific subversion of a bacterial mammalian host by its prokaryotic pathogen.

## 5. CONCLUSIONS

Over the last several years the nascent field of cross-kingdom RNA signaling has undergone significant growth. Immune stimulatory effects of bacterial RNA and the role that they play in eliciting and/or suppressing host protective responses are becoming better understood. The sequence-specific effects of regulatory RNAs add an extra dimension of possibilities for RNA signaling. The corresponding development of RNA sequencing and accompanying bioinformatic pipelines now give us powerful tools to investigate this area further. As a consequence, an appreciation that bacterial RNAs are not just a simple uniform trigger of the non-specific immune system, but rather act as complex multifaceted signals is beginning to emerge. On one side is the differential availability of RNA-recognizing sensors within cells, including in their subcellular localization and the need to distinguish between self and foreign RNA. These define the ability of the host to detect intruding RNA and mount a defensive response. On the other side, bacteria appear to utilize multiple methods for protecting and delivering RNA to the host, ranging from MVs which can deliver their cargo over distance, to the intracellular transfer of pathogen’s RNA between host cell compartments. The further coexistence of bacterial RNA with other virulence factors such as lipopolysaccharide^53^, with which they could simultaneously travel, serves to add a potential extra level of complexity to unraveling host/pathogen RNA interactions and effects on inflammatory responses^128,129^.

To advance knowledge, careful experimental design and data interpretation is required to physiologically model relevant amounts and modes of RNA delivery into human cells. Overall, many important technical and biological questions await answers in the coming years to decipher the messages conveyed to human cells by bacterial RNA. Identification of the human cellular, molecular, and genetic networks that can both interact with, and be manipulated by, bacterial RNA signals offers exciting new research directions in the study of bacterial pathogenesis.

## Bullet Points

◊ **Bacterial RNA can be delivered to human cells during infection**

◊ **Bacterial RNA can have effects beyond simple sensing as a pathogen danger signal**

◊ **Bacterial RNA can act in sequence-independent and sequence-dependent mechanisms**

## Open Questions

◊ **What are the key bacterial RNAs?**

◊ **How important is the effect of RNA when compared to protein virulence factors?**

◊ **Do bacterial RNAs exert an effect in the same way as miRNA?**
